# Risk Factors for Obesity and Overfat among Primary School Children in Mashonaland West Province, Zimbabwe

**DOI:** 10.3390/ijerph15020249

**Published:** 2018-02-02

**Authors:** George Kambondo, Benn Sartorius

**Affiliations:** Discipline of Public Health, School of Nursing and Public Health, University of KwaZulu-Natal, Durban 4001, South Africa; Sartorius@ukzn.ac.za

**Keywords:** school children, obesity, risk factors, Zimbabwe

## Abstract

Associated childhood obesity risk factors are not well established in developing countries such as Zimbabwe and this information is essential for tailored intervention development. This study aimed to identify prominent risk factors for overweight/obese and overfat/obese among primary school children of Mashonaland West Province in Zimbabwe. A school-based cross-sectional study was conducted using multi-stage random cluster sampling approach (30 × 30). Bivariate and multivariable logistic regression was employed and identified the risk factors for overweight/obese and overfat/obese. A total of 974 participants were enrolled in the study. Prominent significant risk factors of overweight/obese after multivariable adjustment were higher socio-economic households; parental diabetes status; and living in Makonde, Zvimba, Sanyati or Mhondoro-Ngezi district as opposed to Hurungwe district. Risk factors for overfat/obese that remained statically significant were children in urban areas (aOR = 3.19, 95% CI: 2.18−4.66, *p* = 0.000), being one child in a household, and parents who have diabetes mellitus. Living in Makonde, Sanyati, and Zvimba district remained associated with overfat/obese compared to Hurungwe district. This study has identified prominent proximal determinants of overweight/obese and overfat/obese among primary school children in Zimbabwe, to better assist policy guidance. Aggressive education on good nutrition activities should be tailored and targeted to most affected urban areas within high-risk districts.

## 1. Introduction

Childhood obesity is an established problem in high income countries and is now becoming a major public health problem, especially in middle-low income countries [1,2,3]. The rapid increase in obesity and being overweight in developing countries is being exacerbated by reduced physical activity and diets rich in refined grains, vegetable oils, caloric sweeteners, and processed foods [4,5]. This nutrition and physical activity transition is mostly being experienced in urban settings [6]. NCD (non-communicable disease) risk factor collaboration recent study reported that the number of girls with obesity increased from 5 million in 1975 to 50 million in 2016, and boys with obesity also increased from 6 million in 1975 to 74 million in 2016 [7]. The study that measured the health effects of obesity in 195 countries over 25 years [8] found that in 2015, globally, 107.7 million children were obese with an overall prevalence of 5.0%. The prevalence of obesity in both girls and boys increased by 20% between 1980 and 2015, especially in countries with a low socio-demographic index [8]. Sub-Saharan Africa, like many other developing regions, is experiencing a dual burden of under- and over-nutrition [9]. A study estimated the prevalence of childhood overweight and obesity in Africa in 2010 to be 8.5%, and an expected increase to 12.7% by 2020 [10]. Global Burden of Disease estimates from 1990 to 2013 in developing countries suggested increasing prevalence of obesity in children from 8.1% to 12.9% for boys and from 8.4% to 13.4% for girls [11,12]. In 2013, the WHO advocated for zero increase in the prevalence of overweight levels among children, and in the prevalence of obesity among adults [13]. The sustainable development goal (SDG) target for obesity for 2030 is to reduce by one-third premature mortality from non-communicable diseases (NCDs) through prevention and treatment [14]. The SDG indicator tracks the share of a country’s population that is overweight or obese, especially in children who often carry obesity into adulthood [15].

Furthermore, childhood obesity appears to continue into adulthood in 70% of cases [16,17,18,19,20]. These conditions lead to an impaired quality of life for a prolonged period and contribute to premature mortality [21]. Overweight/obesity contributed to four million deaths and 120 million disability-adjusted life-years worldwide in the year 2015 [8].

Obesity is a complex and multifactorial condition [21,22,23]. Studies have indicated that direct risk factors of childhood obesity are the imbalances between food intake and energy expenditure, coupled with obesogenic environments that promote low physical activity [24]. A recent study in India concluded that rural to urban migration in developing settings appears to be associated with both positive (higher fruit and vegetable intake) and negative (higher energy and fat intake) dietary changes [25]. Rural-urban migrations experience the environmental changes associated with very rapid urbanization, enabling epidemiological transitions in a short time period. The prevalence of childhood obesity in developing countries is attributed to growing urbanization, the transition towards a high caloric western diet of refined and fast foods, and a sedentary lifestyle [26,27]. Direct risk factors for childhood obesity are low physical activity, excess calorie intake, and the imbalance between the two in rapidly transitioning African settings [28,29].

The prevalence of overfat/obese has been found to be increasing among primary school children in Mashonaland, West province, in Zimbabwe [30], but no studies have been conducted to identify the prominent local risk factors for childhood obesity among primary school children in southern Africa, and specifically in Zimbabwe. An essential step in the prevention and control of childhood overfat/obese is the identification of locally relevant modifiable risk factors. Understanding the fundamental pathways to childhood obesity will assist in the development of efficient policies and effective, preventative interventions against child obesity. Furthermore, risk factor heterogeneity across geographic and socio-economic status (SES) strata further compounds this problem within rapidly transitioning populations. There is growing evidence that childhood obesity can be more effectively averted by interventions than adult obesity; thus, interventions aimed at modifying risk factors to reduce/prevent childhood obesity in developing settings should be considered [31]. This study thus aimed to identify prominent risk factors for overweight/obese and overfat/obese among primary school children in Mashonaland, West Province, in Zimbabwe.

## 2. Methods

### 2.1. Study Setting

A school-based, cross-sectional study was conducted among school-going children aged from 6 to 12 years in Mashonaland, West Province of Zimbabwe with seven administrative districts. The province has a population of 1,449,938 of which 65% reside in rural areas. The province has a total of 707 primary schools, both government run and private. The provincial primary school enrolment was 310,308 in the year 2015. Study Population were Primary school children aged 6–12 years. The study was conducted in September 2015.

### 2.2. Study Sampling and Sample Size

The traditional 30 × 30 multi-stage cluster sampling strategy for nutritional surveys [32,33] to obtain a representative sample of primary school children aged 6 to 12 years across the province was employed. It assumes a design effect of two, and it estimates prevalence with ±5% precision at 95% confidence and an interval width of 10%. Thirty primary schools (“clusters”) were selected at the first stage using Probability Proportional to Size (PPS) across the five districts within the province. Within each selected school, children were randomly selected from each class register and all children selected were invited to participate in the study until the minimum sample size of 30 had been achieved. Thus, the target sample size for this approach was 900 children. Assuming a response rate of 90%, the total sample size was increased to 990.

### 2.3. Data Collection

All data were entered using Epidata version 3.1 software (The EpiData Association, Odense, Denmark) [34] with built-in validation checks and constraints to ensure high data quality.

### 2.4. Anthropometric Measurements

Anthropometric measurements were conducted by trained nurses, environmental health technicians, and a physiotherapist during the morning break and lunch break. The participants’ height, weight, mid upper arm circumference (MUAC), waist circumference, hip circumference, and percent body fat were recorded while observing standard precautions [35,36].

Overweight/obese was defined in the present study using the International obesity task force (IOTF) classification by [37], in which BMI is calculated for age percentiles. Those who had underweight and healthy weight with BMI for age of below 84.9th percentile were classified as having normal weight; overweight classification was at BMI for age of 85th–94.9th percentile; and obese at BMI for age between 95th and 100th percentile [37].

Being overfat/obese was described using the McCarthy body fat reference curves for school children, and the bio-impedance (TANITA SC240MA, Tanita corporation, Tokyo, Japan) analysis scale was used. Overfatness in this study was defined using cut-offs for excess fatness that were age- and sex-specific, and defined as 85th percentile of body fat percentage. A range between 19.5% and 22.7% was defined as overfat for males, and between 23% and 25.2% was defined as overfat for females, respectively, from the [38] reference. The descriptive statistics for body fat are classified as Normal (which includes individuals who were either underfat or healthy), Overfat, and Obese.

The agreement of the two methods using BMI for age percentiles [37] and overfat/obese [38] was evaluated by Kappa (K) statistic and was very high, at 86.55% (expected agreement = 73.29% at *p* < 0.001).

### 2.5. Socio-Demographic Information

A structured questionnaire was used to collect socio-demographic and detailed risk factor information from the participating school children. This was administered by trained research assistants. Various information was collected, such as age group, gender, district, location, religion, educational/occupational status of parents or guardians, socio-economic status, number of siblings, and parental diabetes status.

### 2.6. Statistical Analysis

All statistical analyses were performed using STATA 13.0 (Stata Corp LP, College Station, TX, USA) [39]. Survey weights were incorporated into the analysis using given the complex multistage sampling design. Association between the outcome variables with both obesity based on BMI for age (IOTF) and with overfatness [38] and the categorical variables were compared using a survey weighted chi-square (χ^2^) test or Fisher’s exact test. Odds ratios (OR) and their 95% confidence interval (CI) were estimated. Bivariate and multivariable logistic regression analyses were performed to measure the association of independent variables for childhood overweight/obese and overfat/obese.

A questionnaire was adopted and modified from the Spotlight programme (bridging the gap) [40], the Global school-based health Survey [41], and from a study on an intervention to promote Healthy Eating and Physical Activity in Lebanese School children that was called Health-E-PALS, a pilot cluster, randomized controlled trial [42]. An adjusted *p*-value of <0.05 was deemed statistically significant. Generalized linear regression and mixed model fit was assessed.

### 2.7. Ethics

Ethical clearance was approved by the Biomedical Research Ethics Committee, University of KwaZulu-Natal (BE074/15) and the Medical Research Council of Zimbabwe (MRCZ\A\1972). Written informed consent was obtained from parents or guardians, including written authorization from the Ministry of Primary and Secondary Education and each participating school head. Consent was also obtained from the participants.

Confidentiality of information was ensured through use of anonymised identifiers on all data collection instruments/tools. After each field visit, completed questionnaires were locked in a cabinet at the Provincial medical officers’ offices and were accessible to the researchers only. The electronic database was password-protected.

## 3. Results

A total of 974 children were recruited, i.e., a response rate of 98%. One Grade Seven class from Karoi School was excluded, as the consent forms were issued only to children who were perceived to be obese, which was not in line with the study design. Table 1 presents the results of the descriptive analyses of demographic characteristics of the school children. The average age of the participants was 10 ± 2.1 years. Gender among the study participants was 463 (48%) males and 511 (52%) females. Makonde district had the highest number of respondents 452 (46.4%), and the fewest participants were from the Mhondoro-Ngezi and Sanyati districts, both with 99 (10.2%) participants, respectively. The majority of the respondents, (538 = 55.2%) were from urban schools and 436 (44.8%) were from rural schools. More participants (13.8% in urban areas as compared to 2.3% in rural areas) were obese, and this was statistically significant (*p* < 0.001).

### Socio-Demographic Risk Factors Associated with Childhood Obesity among Primary School Children

Table 2 presents results for the bivariate and multivariable logistic regression analyses between various independent factors associated with an overweight/obese status.

Firstly, following the bivariate analyses, primary school children in Makonde district were 39 times (COR = 38.85, 95% CI: 5.37−281.35, *p* = 0.001) more likely to be overweight/obese as compared to children in Hurungwe district, while in Mhondoro-Ngezi district children were 31 times more likely (COR = 31.42, 95% CI: 4.10–241.05, *p* = 0.001), in Sanyati district children were 34 times more likely (COR = 33.79, 95% CI: 4.42–258.37, *p* = 0.001), and children from Zvimba district were 14 times (COR = 14.41, 95% CI: 1.86–111.54, *p* = 0.011) more likely to be overweight/obese. Attending a school located in an urban area and having two to four siblings in the family were associated with obesity (COR = 2.87, 95% CI: 1.93–4.28, *p* ≤ 0.000) and (COR = 1.78, 95% CI: 1.11–2.86, *p* = 0.008), respectively. Higher SES (COR = 4.91, 95% CI: 3.14–7.69, *p* = 0.001) and having parents with diabetes mellitus (COR = 3.33, 95% CI: 1.57–7.03, *p* = 0.002) were associated with increased odds of being obese. The mother’s educational status being at tertiary level was associated with obesity (COR = 4.91, 95% CI: 1.41−17.13, *p* = 0.013). However, the mother and father being unemployed were associated with reduced odds of being overweight/obese (COR = 0.23, 95% CI: 0.11–0.51, *p* = 0.001) and (COR = 0.04, 95% CI: 0.01–0.28, *p* = 0.001), respectively.

Following the multivariable analyses, children in Makonde district were 27 times more likely (aOR = 27.43, 95% CI: 3.66–205.50, *p* = 0.001) to be overweight/obese compared to those in Hurungwe district, while in Mhondoro-Ngezi district this was 22 times more likely (aOR = 21.81, 95% CI: 2.68–177.27, *p* = 0.004), in Sanyati district it was 27 times more likely (aOR = 26.50, 95% CI: 3.30–212.57, *p* = 0.002), and in Zvimba district it was 11 times (aOR = 11.06, 95% CI: 1.36–89.73, *p* = 0.024) more likely for children to be overweight/obese. Attending school in an urban area remained associated with obesity with aORs of 2.74 (95% CI: 1.83–4.09, *p* ≤ 0.000). Having one child in the family was associated with four times increased odds of overweight/obese (aOR = 3.83, 95% CI: 1.74–8.43, *p* = 0.001). Higher SES and positive parental diabetes mellitus status also remained significant risk factors for overweight/obesity with aORs of 2.03 (95% CI: 1.03–3.98, *p* = 0.038) and 3.12 (95% CI: 1.25–7.83, *p* = 0.015), respectively. However, only the father being unemployed had a 93% reduced odds of being overweight/obese (aOR = 0.07, 95% CI: 0.01–0.54, *p* = 0.011).

Table 3 presents results for bivariate and multivariable regression analyses between various independent risk factors associated with the overfat/obese categorization. Females had an increased chance of being overfat/obese (COR = 1.42, 95% CI: 1.01–2.01, *p* = 0.045). Children in Makonde district were five times more likely to be overfat/obese (COR = 4.52, 95% CI: 2.14–9.55, *p* = 0.001), in Sanyati district this was five times more likely (4.63, 95% CI: 1.94–11.04, *p* = 0.001), and in Zvimba district children were six times (COR = 5.87, 95% CI: 2.64–13.05, *p* = 0.001) more likely to be overfat/obese, respectively, as compared to Hurungwe district. The school being located in an urban area and having two to four siblings in the family resulted in children being three times more likely to be obese (COR = 3.32, 95% CI: 2.27–4.83, *p* ≤ 0.000) and two times more likely to be overfat (COR = 2.02, 95% CI: 1.30–−3.15, *p* = 0.002), respectively. Higher SES (COR = 4.17, 95% CI: 2.75–6.32, *p* = 0.001) and having parents with diabetes mellitus (COR = 3.09, 95% CI: 1.49–6.41, *p* = 0.003) was associated with increased odds of being overfat/obese. The mother’s educational status being at tertiary level was associated with overfat/obesity (COR = 4.03, 95% CI: 1.33–12.27, *p* = 0.014). Mother and father being unemployed was associated with the reduced odds of being overfat/obese (COR = 0.31, 95% CI: 0.16–0.59, *p* = 0.001) and (COR = 0.17, 95% CI: 0.07–0.40, *p* = 0.001), respectively.

Following the multivariable logistic regression, children in Makonde district were three times (aOR = 2.84, 95% CI: 1.24–6.48, *p* = 0.013) more likely to be overfat/obese, in Sanyati district they were three times (aOR = 3.20, 95% CI: 1.22–8.36, *p* = 0.018) more likely, and in Zvimba district they were five times (aOR = 4.71, 95% CI: 1.93–11.50, *p* = 0.001) more likely to be overfat/obese respectively, compared to those Hurungwe district. There was no association between living in Mhondoro-Ngezi district and being overfat/obese. Urban school location and positive parental diabetes status remained significant risk factors for overfat/obese status (aOR = 3.19, 95% CI: 2.18–4.66, *p* ≤ 0.000) and (aOR = 2.85, 95% CI: 1.20–6.76, *p* = 0.018), respectively. Tertiary level paternal education level and self- or unemployed fathers remained independently associated with reduced odds of school children being overfat/obese. Households with one child and two to four children were independently associated with higher odds of overfat/obese with aORs 2.93 (95% CI: 1.32–6.48, *p* = 0.008) and 1.90 (95% CI: 1.21–2.98, *p* = 0.005), respectively.

## 4. Discussion

Few studies have been conducted in southern Africa regarding the risk factors for overweight/overfat among school children, especially in Zimbabwe, where the most significant independent risk factors that were associated with overweight/obese and overfat/obese in primary school children were found in Makonde, Sanyati, and Zvimba districts, an urban location of schools, households with fewer siblings, higher socio-economic status, and a positive parental diabetes status. This study also found that a father’s education being at tertiary level and a father being unemployed had a protective effect on being overfat/obese.

The study did not find any association between gender and age with overweight/obese and overfat/obese, respectively. This is in contrast to other similar studies that have indicated that obesity was associated with age, sex, SES, urbanization, and ethnicity [43]. Other studies also indicate that when obesity and gender difference are evaluated, girls are reported to be affected more than boys [44,45,46,47]. In the present study, gender and age were not associated with overweight/obese and overfat/obese. Makonde, Sanyati, and Zvimba districts had higher risks of both overweight/obese and overfat/obese when compared to Hurungwe district. This may be indicative of nutrition transitions occurring faster throughout Sub-Saharan Africa in the context of rapid urbanization [48]. In addition, a higher SES is important, as these districts are surrounded by urban areas and mines [49]. Socio-economic status has been found to be an important predictor of childhood overweight/obesity, as lower SES has been reported to be an important predictor of obesity in many industrialized [50] and developing countries [51,52,53,54,55,56]. The study finding is consistent with findings in studies conducted in developing countries for SES to be significantly associated with overweight/obese only. In this study, higher socio-economic class showed a significant direct association (*p* = 0.038) with overweight/obese with BMI, but no association with overfat/obese. However, the result contrasts with findings from the United Kingdom that found an association between adiposity and SES in children when a BMI-z score was used as the outcome [25,57].

The present study found that the location of the school being in an urban area was associated with a greater risk of both overweight/obese and overfat/obese as compared to rural schools (*p* ≤ 0.000). The relationship observed may be associated with the increase in a sedentary lifestyle and with the exposure of urban-based children in a higher SES category to processed foods. This finding may be attributed to their exposure to energy-dense processed food and to the fact that there was no walking to school by the majority of school children in urban areas [58]. In urban areas, there are also uncontrolled food and beverage advertisements on print and TV, as compared to rural areas where advertisements are limited [59]. During the data collection, the researcher observed vending of chips at the majority of school premises in urban schools that exposed the children to junk food.

Another interesting finding from the present study was that households with one child were associated with a higher risk of both overweight/obese and overfatness. These results are consistent with other studies in which being an only child was significantly associated with obesity [60,61]. This is understood because when there is only one child in a family, they tend to have fewer opportunities to engage in physical activity and also tend to have higher food and fat intake [62].

The father’s education level being at tertiary level was associated with 71% reduced odds of being overfat/obese (*p* ≤ 0.047) in this study as compared to overweight/obese. These results demonstrated that variables of the father’s higher educational level, being self-employed, and being unemployed reduced the risk of being overfat/obese. The results contradict what was found in another study among South African adolescents in which the household head who had an education less than a secondary level certificate had a protective effect against obesity, while someone with a secondary and tertiary educational level, which may lead to higher SES, was not significantly associated with the children of the household being overweight/obese [49]. These findings are contrary to Indian and Ghanaian studies that reported maternal unemployment to be a risk factor for childhood overweight/obese [63,64]. These results are mixed, as in developing countries a higher educational level is usually associated with being overweight/obese and overfat/obese, as the parent is rated to be in a higher SES with the ability to purchase sufficient food. These results are inverse to those found in developed countries where a higher educational level of the father, and a mother who is unemployed or in low-earning occupations, were associated with a higher risk of being overweight/obese [65]. This study suggests that unemployment had a protective effect on those children who were overweight/obese and overfat/obese, as there was no significant association between the mother’s employment and her level of education. This finding is contrary to other studies that found an increased risk of overweight among children of mothers with a higher educational level in developing countries [66,67]. This finding has been revealed by overfat/obese as an outcome as compared to overweight/obese, and this association was not detected in this study [54].

The study found an independent, significant association of maternal and parental diabetes status with both overweight/obese and overfat/obese categories. These results are consistent with many studies conducted, which concluded that development in a diabetic intrauterine environment results in excess fetal growth as maternal glucose freely crosses the placenta [68,69,70]. A study with Indian women found that offspring of mothers with pre-existent, type 2 diabetes or gestational diabetes mellitus were larger for gestational age at birth and at every age were heavier than the offspring of pre-diabetic or non-diabetic women [71,72,73]. There is a need to evaluate the effects of exposure to diabetes *in utero* on childhood growth and body size among children, as it is critical for the development of interventions for offspring adiposity. Maternal glucose-insulin metabolism can lead to an increased risk of childhood obesity, and this thus becomes a vicious cycle of obesity from one generation to the next [74].

## 5. Study Limitations

This study had several limitations. The study did not assess other risk factors for childhood obesity, which include maternal smoking, no or short-term breast feeding, infant size and growth, weight-gain during pregnancy, and maternal obesity [75]. Further research is recommended to assess the relationship of unhealthy dietary patterns and physical inactivity with risk factors such as TV exposure, insufficient sleep, consumption of sugar-sweetened beverages, daily physical activity of less than 30 min duration, and mode of transport to school. The present research is a cross-sectional study; therefore, a causal relationship cannot be inferred, although the risk factors have partly predicted the onset of obesity among school children.

## 6. Conclusions

In conclusion, the most justifiable target school districts in Mashonaland West for school children needing overweight/obese and overfat/obese prevention interventions are Makonde, Zvimba, and Sanyati, with the exception of Mhondoro-Ngezi district. We recommend that schools located in urban areas are given priority on interventions. Secondly, health education programmes addressing childhood obesity and risk factors should start at an early age in schools. Furthermore, these findings indicate a need for policy makers to consider childhood obesity in school children to be a crisis. This would therefore make it a funded government and public health priority, which could join forces across the disciplines of health professionals, educationists, and families to mount an effective public campaign in the prevention of obesity and the implementation of school and family based interventions. Finally, continuous and accurate further research of the evolving situation will allow us to provide relevant tools and strategies for health promotion.

## Figures and Tables

**Table 1:** Descriptive characteristics of overweight/obese and overfat/obese study of school children, as defined by IOTF (Cole, 2012), BMI-for-age reference, and (McCarthy, 2006) body fat reference [37,38].

Characteristics: *n* = 974 (% ^i^)			Overweight/Obese Defined from BMI with IOTF Reference				Overfat/Obese Defined from Bioelectrical Impedance with the McCarthy Reference			
	Category	Total (*n* = 974) ^ii^	Normal (N = 840)	Overweight (N = 59)	Obese (N = 75)	*p*-Value	Normal (N = 815)	Overfat (N = 75)	Obese (N = 84)	*p*-Value
Age group	6 years	19 (1.95)	14 (73.68)	3 (15.79)	2 (10.53)	0.013	17 (89.47)	1 (5.26)	1 (5.26)	0.001
	7–9 years	246 (25.26)	200 (81.3)	18 (7.32)	28 (11.38)		185 (75.2)	26 (10.57)	35 (14.23)	
	10–12 years	709 (72.79)	626 (88.29)	38 (5.36)	45 (6.35)		613 (86.46)	48 (6.77)	48 (6.77)	
Gender	Male	463 (47.54)	402 (86.83)	27 (5.83)	34 (7.34)	0.88	399 (86.18)	33 (7.13)	31 (6.7)	0.089
	Female	511 (52.46)	438 (85.71)	32 (6.26)	41 (8.02)		416 (81.41)	42 (8.22)	53 (10.37)	
District	Hurungwe	164 (16.84)	163 (99.39)	0 (0)	1 (0.61)	0.001	156 (95.12)	7 (4.27)	1 (0.61)	<0.001
	Makonde	452 (46.41)	365 (80.75)	38 (8.41)	49 (10.84)		367 (81.19)	25 (5.53)	60 (13.27)	
	Mhondoro-Ngezi	99 (10.16)	83 (83.84)	9 (9.09)	7 (7.07)		89 (89.9)	6 (6.06)	4 (4.04)	
	Sanyati	99 (10.16)	82 (82.83)	8 (8.08)	9 (9.09)		80 (80.81)	12 (12.12)	7 (7.07)	
	Zvimba	160 (16.43)	147 (91.88)	4 (2.5)	9 (5.63)		123 (76.88)	25 (15.63)	12 (7.5)	
Location	Urban	538 (55.24)	426 (79.18)	42 (7.81)	70 (13.01)	0.001	412 (76.58)	52 (9.67)	74 (13.75)	<0.001
	Rural	436 (44.76)	414(94.95)	17 (3.9)	5 (1.15)		403 (92.43)	23(5.28)	10 (2.29)	
Religion	None	40 (4.11)	36 (90)	4 (10)	0 (0)	0.008	37 (92.5)	3 (7.5)	0 (0)	<0.001
	Catholic	85 (8.73)	71 (83.53)	5 (5.88)	9 (10.59)		68 (80)	7 (8.24)	10 (11.76)	
	Protestant	265 (27.21)	221 (83.4)	15 (5.66)	29 (10.94)		217 (81.89)	21 (7.92)	27 (10.19)	
	Traditional	18 (1.85)	13 (72.22)	1 (5.56)	4 (22.22)		10 (55.56)	1 (5.56)	7 (38.89)	
	Apostolic	295 (30.29)	270 (91.53)	15 (5.08)	10 (3.39)		265 (89.83)	14 (4.75)	16 (5.42)	
	Pentecostal	271 (27.82)	229 (84.5)	19 (7.01)	23 (8.49)		218 (80.44)	29 (10.7)	24 (8.86)	
Mother’s education	None	35 (3.59)	32 (91.43)	1 (2.86)	2 (5.71)	0.001	31 (88.57)	2 (5.71)	2 (5.71)	<0.001
	Primary	144 (14.78)	130 (90.28)	7 (4.86)	7 (4.86)		126 (87.5)	11 (7.64)	7 (4.86)	
	Secondary	665 (68.28)	583 (87.67)	41 (6.17)	41 (6.17)		566 (85.11)	49 (7.37)	50 (7.52)	
	Tertiary	111 (11.4)	76 (68.47)	10 (9.01)	25 (22.52)		73 (65.77)	13 (11.71)	25 (22.52)	
Mother’s occupation	Formally employed	231 (23.72)	182 (78.79)	18 (7.79)	31 (13.42)	0.001	172 (74.46)	26 (11.26)	33 (14.29)	0.002
	Self employed	228 (23.41)	204 (89.47)	13 (5.7)	11 (4.82)		194 (85.09)	19 (8.33)	15 (6.58)	
	Unemployed	136 (13.96)	128 (94.12)	3 (2.21)	5 (3.68)		123 (90.44)	6 (4.41)	7 (5.15)	
	House wife	360 (36.96)	307 (85.28)	25 (6.94)	28 (7.78)		307 (85.28)	24 (6.67)	29 (8.06)	
Father’s educational level	None	23 (2.36)	20 (86.96)	0 (0)	3 (13.04)	0.001	17 (73.91)	3 (13.04)	3 (13.04)	0.005
	Primary	53 (5.44)	52 (98.11)	1 (1.89)	0 (0)		49 (92.45)	4 (7.55)	0 (0)	
	Secondary	683 (70.12)	604 (88.43)	38 (5.56)	41 (6)		584 (85.51)	47 (6.88)	52 (7.61)	
	Tertiary	165 (16.94)	125 (75.76)	15 (9.09)	25 (15.15)		123 (74.55)	18 (10.91)	24 (14.55)	
	Died	50 (5.13)	39 (78)	5 (10)	6 (12)		42 (84)	3 (6)	5 (10)	
Father’s occupation	Formally employed	486 (49.9)	401 (82.51)	36 (7.41)	49 (10.08)	0.001	375 (77.16)	52 (10.7)	59 (12.14)	<0.001
	Self employed	313 (32.14)	276 (88.18)	17 (5.43)	20 (6.39)		279 (89.14)	14 (4.47)	20 (6.39)	
	Unemployed	125 (12.83)	124 (99.2)	1 (0.8)	0 (0)		119 (95.2)	6 (4.8)	0 (0)	
	Died	50 (5.13)	39 (78)	5 (10)	6 (12)		42 (84)	3 (6)	5 (10)	
Number of siblings	One	43 (4.41)	30 (69.77)	4 (9.3)	9 (20.93)	0.002	31 (72.09)	5 (11.63)	7 (16.28)	0.006
	Two–four	659 (67.66)	562 (85.28)	45 (6.83)	52 (7.89)		539 (81.79)	56 (8.5)	64 (9.71)	
	>5	272 (27.93)	248 (91.18)	10 (3.68)	14 (5.15)		245 (90.07)	14 (5.15)	13 (4.78)	
Socio-economic status	≤$200 Low	547 (56.16)	503 (91.96)	21 (3.84)	23 (4.2)	0.001	488 (89.21)	35 (6.4)	24 (4.39)	<0.001
	$201–$500 Medium	254 (26.08)	216 (85.04)	20 (7.87)	18 (7.09)		212 (83.46)	17 (6.69)	25 (9.84)	
	≥$501	173 (17.76)	121 (69.94)	18 (10.4)	34 (19.65)		115 (66.47)	23 (13.29)	35 (20.23)	
Mother’s vital status	Alive	955 (98.05)	821 (85.97)	59 (6.18)	75 (7.85)	0.429	796 (83.35)	75 (7.85)	84 (8.8)	0.197
	Dead	19 (1.95)	19 (100)	0 (0)	0 (0)		19 (100)	0 (0)	0 (0)	
Parental Diabetes status	No	941 (96.61)	818 (86.93)	55 (5.84)	68 (7.23)	0.004	794 (84.38)	73 (7.76)	74 (7.86)	0.001
	Yes	33 (3.39)	22 (66.67)	4 (12.12)	7 (21.21)		21 (63.64)	2 (6.06)	10 (30.3)	

^i^: row percentage; ^ii^: column percentage: *p* < 0.05.

**Table 2:** Bivariate and multivariable regression analysis of primary school children demographics to analyze risk of overweight/obese, defined from BMI with IOTF (Cole, 2012) and BMI-for-age reference [37].

Overweight/Obese Regression Analysis		Bivariate		Multivariable	
Characteristics: *n* = 974	Category	COR (95% CI)	*p*-Value	aOR (95% CI)	*p*-Value
Age group	6 years	1 (ref)		1 (ref)	
	7–9 years	0.64 (0.22–1.88)	0.42	0.64(0.19–2.80)	0.642
	10–12 years	0.37 (0.13–1.06)	0.063	0.33(0.09–1.26)	0.106
Gender	Male	1 (ref)		1 (ref)	
	Female	1.1 (0.76–1.58)	0.615	1.06(0.70–1.60)	0.767
District	Hurungwe	1 (ref)		1 (ref)	
	Makonde	38.85 (5.37–281.35)	0.001	27.43(3.66–205.50)	0.001
	Mhondoro-Ngezi	31.42 (4.1–241.05)	0.001	21.81(2.68–177.27)	0.004
	Sanyati	33.79 (4.42–258.37)	0.001	26.50(3.30–212.57)	0.002
	Zvimba	14.41 (1.86–111.54)	0.011	11.06(1.36–89.73)	0.024
Location	Rural	1 (ref)		1 (ref)	
	Urban	2.87 (1.93–4.28)	0.00	2.74(1.83–4.09)	0.000
Religion	None	1 (ref)		1 (ref)	
	Catholic	1.77 (0.54–5.78)	0.341	0.77(0.19–3.12)	0.719
	Protestant	1.79 (0.61–5.29)	0.291	0.64(0.18–2.30)	0.500
	Traditional	3.46 (0.8–14.9)	0.095	1.64(0.27–9.78)	0.588
	Apostolic	0.83 (0.27–2.53)	0.748	0.53(0.14–1.93)	0.333
	Pentecostal	1.65 (0.56–4.88)	0.365	0.76(0.21–2.75)	0.678
Mother’s educational level	None	1 (ref)		1 (ref)	
	Primary	1.15 (0.31–4.24)	0.835	1.23(0.28–5.33)	0.782
	Secondary	1.5 (0.45–5.01)	0.51	1.00(0.26–3.93)	0.998
	Tertiary	4.91 (1.41–17.13)	0.013	1.14(0.24–5.34)	0.865
Mother’s occupation	Formally employed	1 (ref)		1 (ref)	
	Self employed	0.44 (0.26–0.74)	0.002	0.85 (0.41–1.77)	0.667
	Unemployed	0.23 (0.11–0.51)	0.001	0.54(0.21–1.36)	0.193
	House wife	0.64 (0.42–0.99)	0.043	1.33 (0.71–2.50)	0.372
Father’s educational level	None	1 (ref)		1 (ref)	
	Primary	0.13 (0.01–1.31)	0.083	0.12 (0.01–1.50)	0.100
	Secondary	0.87 (0.25–3)	0.828	0.62 (0.14–2.72)	0.528
	Tertiary	2.13 (0.6–7.56)	0.24	0.62 (0.13–2.91)	0.548
Father’s occupation	Formally employed	1 (ref)		1 (ref)	
	Self employed	0.63 (0.42–0.96)	0.031	1.06(0.63–1.76)	0.832
	Unemployed	0.04 (0.01–0.28)	0.001	0.07(0.01–0.54)	0.011
Number of siblings in the family	>5	1 (ref)		1 (ref)	
	two -four	1.78(1.11–2.86)	0.008	1.68(1.04–2.70)	0.033
	One	4.48(2.06–9.71)	0.016	3.83(1.74–8.43)	0.001
Socio-economic status	≤$200 Low	1 (ref)		1 (ref)	
	$201-$500 Medium	2.01 (1.27–3.19)	0.003	0.96(0.55–1.68)	0.893
	≥$501	4.91 (3.14–7.69)	0.001	2.03(1.03–3.96)	0.038
Mother’s vital status	Alive	1 (ref)		1 (ref)	
	Dead			omitted	omitted
Parental Diabetes Status	No	1 (ref)		1 (ref)	
	Yes	3.33 (1.57–7.03)	0.002	3.12(1.25–7.83)	0.015

**Table 3:** Bivariate and multivariable regression analysis of primary school children demographics to analyze risk of overfat/obese, defined from Bioelectrical Impedance measures with the McCarthy et al. 2006 body fat-for-age reference [38].

Overfat/Obese Regression Analysis		Bivariate		Multivariable	
Characteristics: *n* = 974	Category	COR (95% CI)	*p*-Value	aOR (95% CI)	*p*-Value
Age group	6 years	1 (ref)		1 (ref)	
	7–9 years	2.8 (0.63–12.48)	0.176	3.76(0.71–19.85)	0.119
	10–12 years	1.33 (0.3–5.85)	0.705	1.27(0.25–6.57)	0.776
Gender	Male	1 (ref)		1 (ref)	
	Female	1.42 (1.01–2.01)	0.045	1.31(0.89–1.93)	0.172
District	Hurungwe	1 (ref)		1 (ref)	
	Makonde	4.52 (2.14–9.55)	0.001	2.84(1.24–6.48)	0.013
	Mhondoro-Ngezi	2.19 (0.83–5.75)	0.111	1.27(0.44–3.71)	0.659
	Sanyati	4.63 (1.94–11.04)	0.001	3.20(1.22–8.36)	0.018
	Zvimba	5.87 (2.64–13.05)	0.001	4.71(1.93–11.50)	0.001
Location	Rural	1 (ref)		1 (ref)	
	Urban	3.32(2.27-4.83)	0.000	3.19(2.18–4.66)	0.000
Religion	None	1 (ref)		1 (ref)	
	Catholic	3.08 (0.85–11.21)	0.087	1.93(0.47–8.03)	0.363
	Protestant	2.73 (0.81–9.22)	0.106	1.51(0.39–5.79)	0.551
	Traditional	9.87 (2.2–44.2)	0.003	4.56(0.82–25.55)	0.084
	Apostolic	1.4 (0.41-4.8)	0.597	1.08(0.28–4.19)	0.911
	Pentecostal	3 (0.89-10.1)	0.076	1.88(0.49–7.24)	0.357
Mother’s educational level	None	1 (ref)		1 (ref)	
	Primary	1.11 (0.35–3.51)	0.863	1.48(0.41–5.35)	0.548
	Secondary	1.36 (0.47–3.92)	0.575	1.12(0.34–3.71)	0.849
	Tertiary	4.03 (1.33–12.27)	0.014	1.32(0.33–5.20)	0.694
Mother’s occupation	Formally employed	1 (ref)		1 (ref)	
	Self employed	0.51 (0.32–0.82)	0.005	1.14(0.59–2.18)	0.699
	Unemployed	0.31 (0.16–0.59)	0.001	0.56(0.25–1.23)	0.149
	House wife	0.5 (0.33–0.76)	0.001	1.12(0.63–1.10)	0.699
Father’s educational level	None	1 (ref)		1 (ref)	
	Primary	0.23 (0.06-0.92)	0.038	0.22(0.05–1.02)	0.053
	Secondary	0.48 (0.18–1.25)	0.132	0.36(0.12–1.09)	0.071
	Tertiary	0.97 (0.36–2.62)	0.948	0.29(0.09–0.98)	0.047
Father’s occupation	Formally employed	1 (ref)		1 (ref)	
	Self employed	0.41 (0.27–0.62)	0.001	0.43(0.26–0.72)	0.001
	Unemployed	0.17 (0.07–0.4)	0.001	0.21(0.08–0.56)	0.002
Number of siblings in the family	>5	1 (ref)		1 (ref)	
	Two –four	2.02(1.30–3.15)	0.002	1.90(1.21–2.98)	0.005
	One	3.51(1.62–7.63)	0.002	2.93(1.32–6.48)	0.008
Socio-economic status	≤$200 Low	1 (ref)		1 (ref)	
	$201–$500 Medium	1.64 (1.07–2.51)	0.023	0.79(0.47–1.33)	0.379
	≥$501	4.17 (2.75–6.32)	0.001	1.62(0.88–2.99)	0.125
Mother’s vital status	Alive	1 (ref)		1 (ref)	
	Dead		0	omitted	
Diabetes Mellitus status of parents	No	1 (ref)		1 (ref)	
	Yes	3.09 (1.49–6.41)	0.003	2.85(1.20–6.76)	0.018
